# Clinical improvement in a patient with monostotic melorheostosis after treatment with denosumab: a case report

**DOI:** 10.1186/s13256-018-1820-y

**Published:** 2018-09-27

**Authors:** Sarah Byberg, Bo Abrahamsen, Moustapha Kassem, Stuart Ralston, Peter Schwarz

**Affiliations:** 1 Department of Endocrinology, Copenhagen University Hospital Rigshospitalet, Blegdamsvej 9, 2100 Copenhagen, Denmark; 2 Department of Medicine, Holbæk Hospital, Denmark and OPEN, Institute of Clinical Research, University of Southern Denmark, Odense, Denmark; 3 Department of Endocrinology and Metabolism, Odense University Hospital, Odense, Denmark; 4 Rheumatology and Bone Diseases Unit, Western General Hospital, University of Edinburgh, Edinburgh, UK

**Keywords:** Melorheostosis, Denosumab, Case report

## Abstract

**Background:**

A 20-year-old Danish woman with melorheostosis in her right femoral shaft and disabling pain in the affected area, whose symptoms did not in the long term respond to zoledronic acid, experienced continuous remission of pain after treatment with denosumab. To the best of our knowledge, this is the first case report on denosumab treatment for melorheostosis.

**Case presentation:**

Radiologic findings and bone biopsy showed irregular cortical hyperostosis in the right femoral shaft with increased tracer uptake on Tc^99^-bone scan.

The diagnosis of melorheostosis was made based on the radiological findings. There was a good initial response to zoledronic acid administration, but after relapse of pain, the second and third administrations had a poor effect. As a second line of treatment denosumab was administered at 8-week intervals, the frequency was based on our patient’s symptoms and on biochemical markers of bone turnover.

**Conclusion:**

This is the first report indicating that denosumab has a place in the treatment of melorheostosis when the effect of bisphosphonate treatment is insufficient.

## Background

Melorheostosis is a rare, nonhereditary sclerosing bone dysplasia first described in 1922 by Leri and Joanny [1]. The minimal prevalence has been estimated to be 0.9 per million [2]. Establishing the diagnosis melorheostosis is challenging. It is primarily based on radiological findings as defined by Freyschmidt [3] and on the exclusion of other sclerosing bone diseases. It is a chronic progressive disorder and affects females and males equally [3] with no known curative treatment [4, 5]. Case reports have documented pain relief by treatment with bisphosphonates [6–11].

This is the first report describing the treatment of melorheostosis with denosumab. Denosumab was introduced as a second line of treatment, after insufficient effect of zoledronic acid. Treatment with denosumab has shown remarkable clinical effect.

## Case presentation

A 20-year-old Danish woman was referred with constant pain in the right proximal thigh that had progressed for 8 years. She had no other known medical conditions. Menarche was at age 13. There was no family history of sclerosing bone diseases. The limb pain was aggravated at night and was moderately relieved during physical activity and by cold baths. She was on daily analgesic medication with nonsteroidal anti-inflammatory drugs, paracetamol, and weak opioid agonists, and she was on sick leave from her university studies at the time of referral due to her symptoms. She was using shoe inserts because of leg length discrepancy.

At age 11 she had consulted a rheumatologist because of hip joint pain. Guided by ultrasound a hip joint puncture was performed due to fluid gathering; serology was negative. After the puncture, the joint pain ceased.

Six months prior to referral to our clinic, she was involved in a traffic accident and hit by a car from the right at knee level. She was discharged from an emergency room (ER) with no suspected fracture and a radiological examination was not performed. She reported that there was significant worsening of the femoral pain after the traffic accident.

### Clinical findings

A clinical examination revealed no skin lesions, *café au lait* spots, or redness; there was no increased temperature or swelling in her right thigh. Leg length was unequal but within normality.

### Diagnostic assessment

She was instructed to fill out a diary with daily registration of pain on an analog scale from zero to 10, where increasing values were equivalent to more intensive pain. Orally administered analgesics were required when the pain exceeded a score of 6 or more, and the score 10 was reserved for symptoms that kept our patient from falling asleep and that did not respond adequately to orally administered analgesics.

**Fig. 1:**
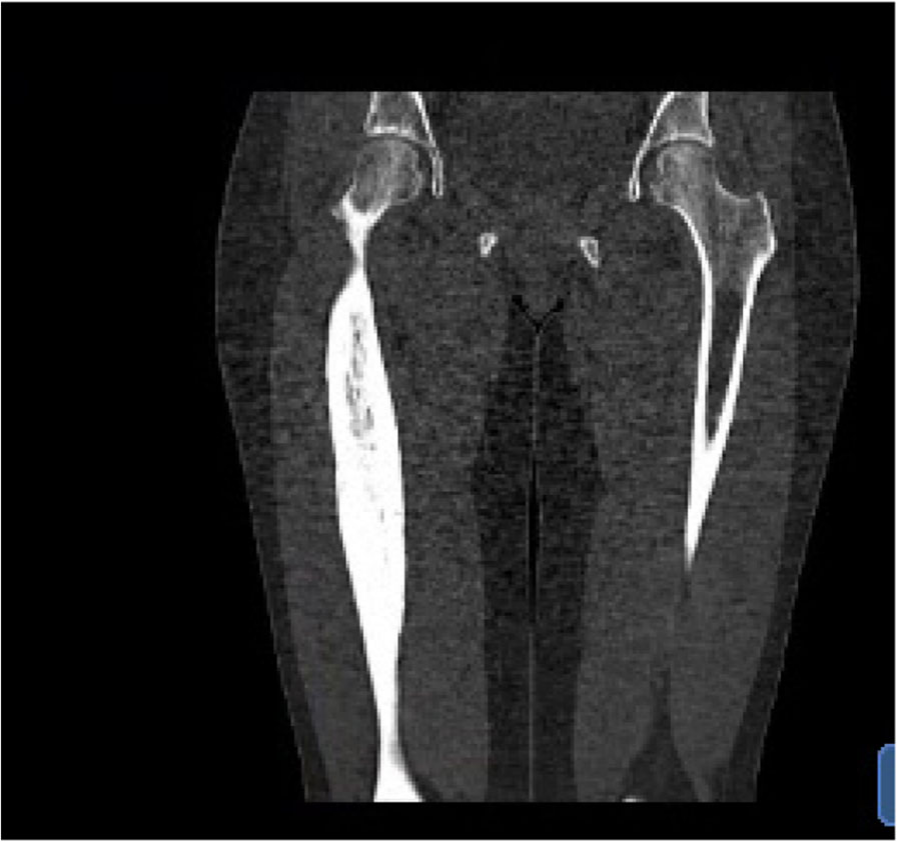
Computed tomography of right femoral shaft. Computed tomography scan showing cortical thickening of the patien’s right femoral shaft

**Fig. 2:**
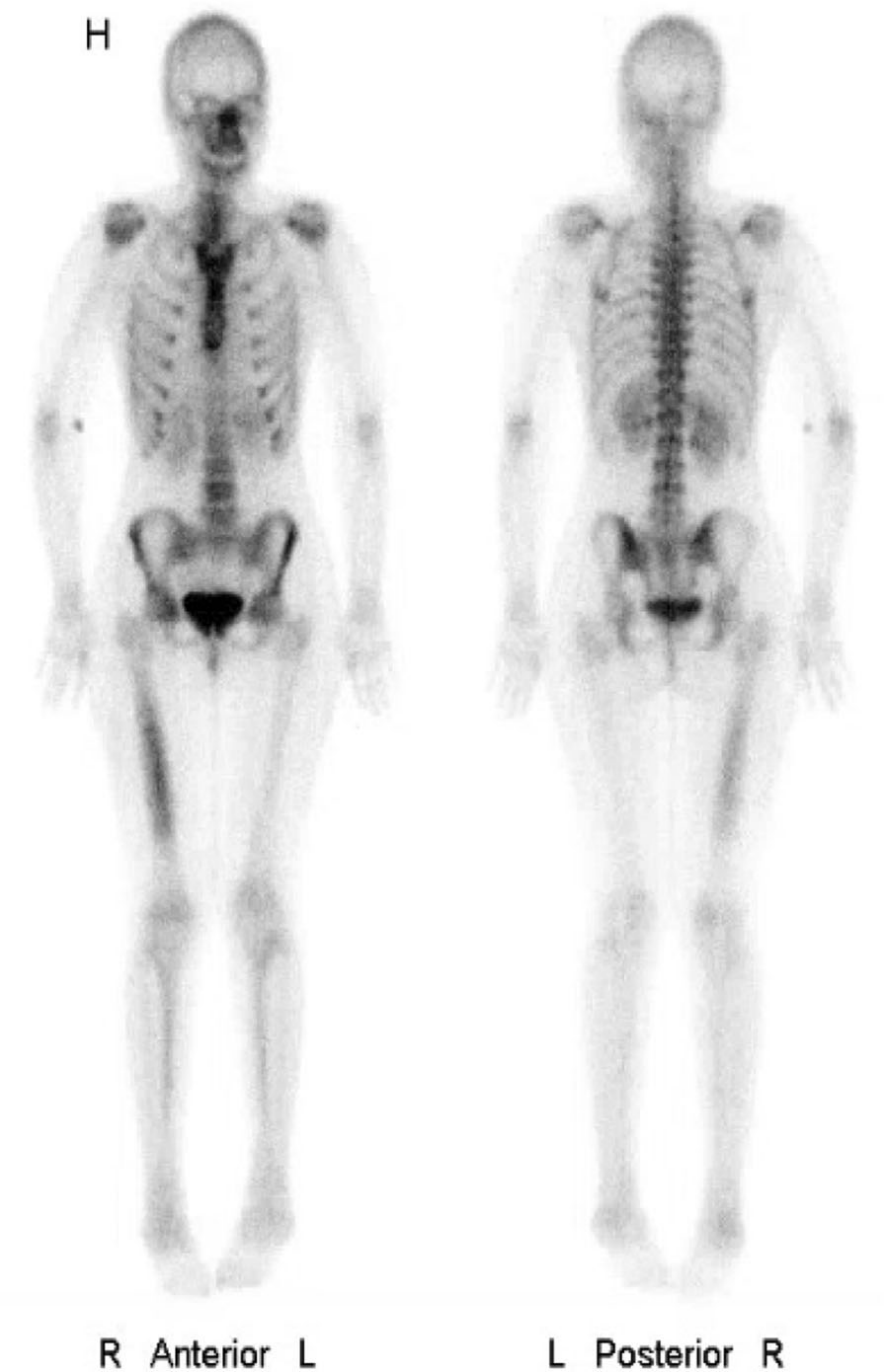
Bone scintigraphy whole body. Bone scintigraphy showing the patient’s increased tracer uptake in the right femoral shaft

Magnetic resonance (MR) and positron emission tomography-computed tomography (PET-CT) scans revealed significant increased cortical thickness in the right femoral diaphysis that partially obliterated the medullary cavity (Fig. 1). The surface of the sclerotic bone was uneven. A Tc^99^-bone scan showed a pathological increase in uptake in most of the right femoral shaft (Fig. 2).

Biochemical bone markers were normal, including normal alkaline phosphatase.

Screening for the GNAS1 activating mutation R201 was negative in peripheral blood; as was mutation screening of the *SQSTM1* gene, which has been reported to be mutated in Paget’s disease of bone.

A bone biopsy was performed and showed thickened bone springs with preserved lamellar structure and slightly accentuated cement lines. There was no fibrosis, inflammation, or increased alcian positivity, which would be suggestive of active osteomyelitis. There were no signs of malignancy or neoplasia.

A gynecological examination prior to referral had found a mild degree of polycystic ovaries (PCO) with normal androgen status. There were no endocrinopathies.

### Diagnostic reasoning and differential diagnosis

During the clinical investigation, four tentative diagnoses were raised: atypical fibrous dysplasia, Paget’s disease of bone, chronic non-infectious osteomyelitis, and melorheostosis. The young age of our patient, normal alkaline phosphatase, and sparing of the metaphysis of the affected bone made Paget’s disease of bone highly unlikely.

**Fig. 3:**
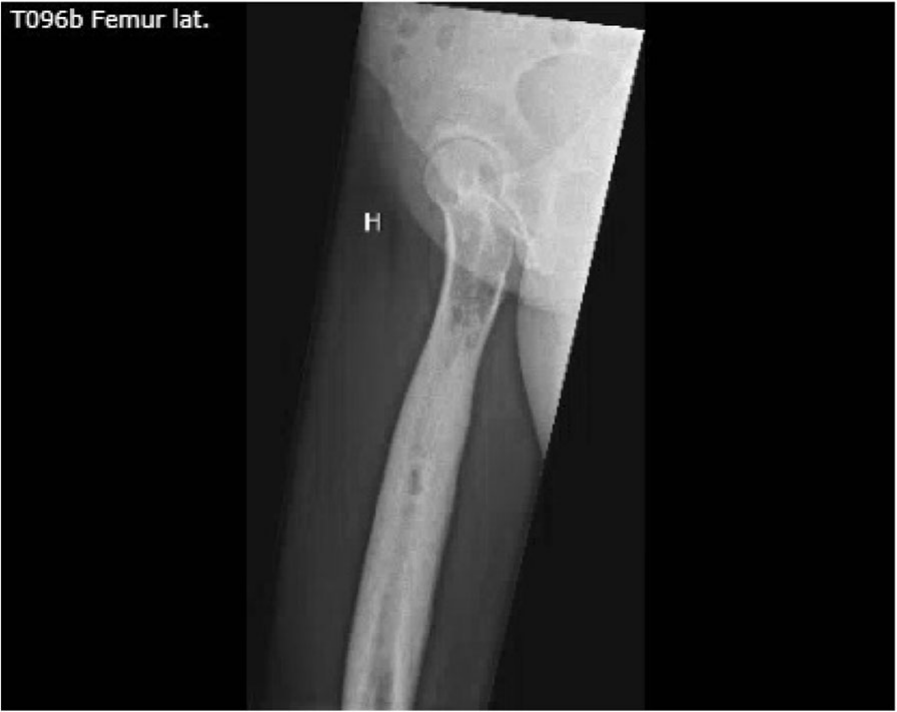
X-ray right femoral shaft. X-ray of the patient’s right femoral shaft showing irregular hyperostosis

The cortical thickening is atypical for the diagnosis of fibrous dysplasia. The absence of *café au lait* spots, the normal age at menarche, the absence of other endocrine disorders, and the negative GNAS1 mutation examination weighed against a diagnosis of fibrous dysplasia. The long bones are a common location for chronic non-infectious osteomyelitis and this can present in childhood; however, the radiological appearance and bone histology did not provide support for this differential diagnosis. With special emphasis on the uneven surface of the affected bone on X-ray (Fig. 3), the diagnosis of melorheostosis, which was fully compatible with the relatively unremarkable histology, was found to be the most plausible diagnosis. In addition, the normal bone chemistry is in keeping with melorheostosis [5] although in polyostotic cases alkaline phosphatase can be increased [11].

### Therapeutic intervention

**Fig. 4:**
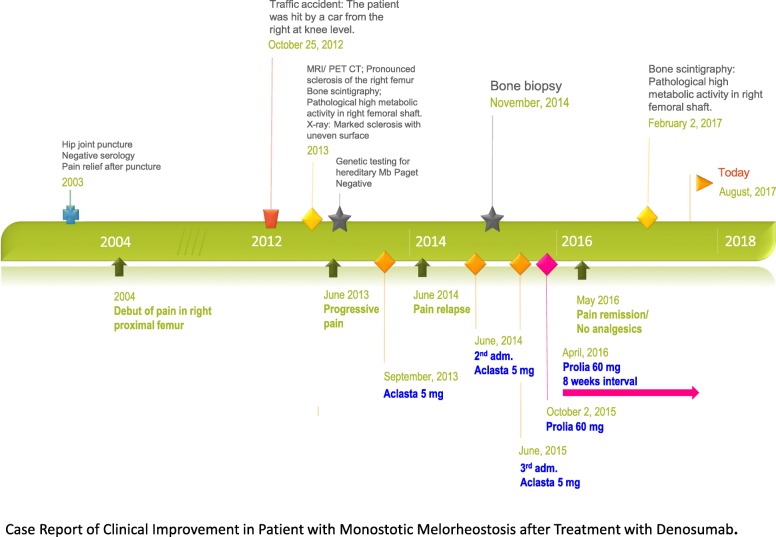
Timeline. Relevant data from this episode of care organized as a timeline. *MRI* magnetic resonance imaging, *PET-CT* positron emission tomography-computed tomography

The timeline for intervention and clinical outcome is presented in Fig. 4. Initially, zoledronic acid 5 mg was administered intravenously three times with 10-month and 12-month intervals; our patient had pain relief after the first administration, but a poor effect of the second and third injection. Using an analog pain scale she reported average pain of 9 prior to treatment, 6 after treatment with zoledronic acid, and 4 from 2 to 10 months after initiation of treatment with denosumab. Looking at the usage of pain medication, it went from daily dosage before treatment to 23% of days after treatment with zoledronic acid, and to no orally administered analgesics for 8 months following treatment with denosumab.

In October 2015, denosumab was chosen as the second line of treatment based on the hypothesis that the pain was caused by increased bone turnover. A dose of 60 mg was injected subcutaneously with some remission of pain that lasted for 6 weeks. At the same time as the pain relapsed, there was an increase in alkaline phosphatase, PINP, CTx, and osteocalcin in blood tests. Biochemistry before and during treatment is listed in Table 1. Based on these findings it was decided to shorten the interval between administrations to further strengthen control of a hypermetabolic state. The second dose of 60 mg was given after 8 weeks and this interval has been kept up to the present.

**Table 1:** Biochemistry before and during treatment with zoledronic acid and denosumab

		Before treatment	After treatment Zoledronic acid	After treatment denosumab	Treatment denosumab	Treatment denosumab
		3 Jul 2013	24 Sep 2013	28 Dec 2015	23 Mar 2016	03 Feb 2017
P-Calcium	mmol/L	2.27		2.32	2.33	2.49
P-Ionized calcium	mmol/L			1.22	1.2	1.23
P-Alkaline phosphatase	U/L	58		43	33	42
P-Alkaline phosphatase, bone type	U/L		5.6	7.2	6.2	7.1
P-Osteocalcin	μg/ L		6.5	9.2	7	5
P-Parathyrin (PTH)	pmol/L			5.23	6.15	8.8
P-25-hydroxy-vitamin D	nmol/L	207		140		98
CTx	pmol/L		< 0.03	< 0.03	< 0.03	< 0.03
PINP	μg/L		12	15.6	11	11.4
Plasma (P)						
			Jun 2015 to Oct 2015	Oct 2015 to Dec 2015	Feb 2015 to Mar 2016	
Mean VAS score		–	–	4	4.3	4.5
Mean VAS (full year)		8	8	6	5	4
% days with pain killers			23	36		0

*PTH* parathyroid hormone, *VAS* visual analog scale

Biochemistry after treatment with both zoledronic acid and denosumab showed suppression of bone-type basic phosphatase, osteocalcin, collagen I, and procollagen I, with a relative increase in parathyroid hormone, and plasma ionized calcium within the normal range. The injections with denosumab were well tolerated with no adverse effects reported. During the whole treatment period, the injections were administered from our out-patient clinic without cancellations.

### Follow-up and outcomes

After the first three doses given 8 weeks apart, there was pain remission for 8 months; in this period our patient did not take any orally administered analgesics. At the latest follow-up in February 2018, she complained of a slight increase in pain and occasional use of paracetamol and nonsteroidal anti-inflammatory drugs.

The treatment with denosumab was well tolerated with no side effects observed.

A Tc^99^-bone scan and computed tomography (CT) scan were performed in February 2017 and in February 2018, 5 and 17 months after the first injection with denosumab; the pathologically increased activity and cortical thickening were stationary.

## Discussion

Melorheostosis occurs sporadically [4]. The disease tends to follow a sclerotomal distribution suggesting it originates in a segmentary embryogenetic defect [12]. This case report describes a case of monostotic sclerosing bone disease in an otherwise healthy young woman. The diagnosis of melorheostosis is based on the radiological findings of uneven hyperostosis of the proximal femur, also known as a flowing candlewax configuration.

Tentative diagnoses have been excluded by: thorough investigation of medical history; clinical, radiological, and biochemical examinations; genetic testing; as well as a biopsy of the affected bone. The bone biopsy was informative only in the sense that it helped exclude active inflammation, malignancy, and fibrous dysplasia. Melorheostosis has been associated with osteogenic sarcoma [13, 14] and malignant fibrous histiocytoma [15]. Histomorphometric evaluation of a bone biopsy measuring 1 cm^3^ showed no sign of malignancy.

In this case report, pain is the only symptom. In other studies of melorheostosis, pain has also been found to be the most prevalent symptom [3]. Melorheostosis can be monostotic or polyostotic and the lower limbs are most often affected [3, 16], in some cases causing a difference in limb length. Associated findings have been reported to be soft tissue fibrosis in the affected area [17], deformity, edema [11], and, rarely, vascular malformations [18]. These are reported in a minority of cases and in the current case there were no associated findings.

It has been speculated whether loss-of-function mutations in *LEMD3* could be central to the pathophysiology of melorheostosis; this mutation is usually not present in cases of sporadic melorheostosis [19]. One research group found that a number of genes coding for adhesion proteins were downregulated in melorheostosis-related skin lesions, with transforming growth factor-β (TGF-β)-induced gene product (βig-h3) being the most significantly affected [20].

In the medical literature, symptomatic relief has been achieved with orthopedic surgery, analgesics, physical therapy, and bisphosphonates [16]. This paper is the first to document that treatment with denosumab has a positive effect on disabling pain in a patient with melorheostosis. The attenuating effect of denosumab on biochemical markers of bone formation and on clinical symptoms has been shown to last approximately 2 months. This may help to shed light on the pathophysiology of a rare disease and points to a high bone turnover. Denosumab was more effective for long lasting pain relief than bisphosphonate in this case report; therefore, one can speculate whether the disease may be caused by local dysregulation in the RANK/RANKL pathway.

A hypermetabolic state is also evident by the increased tracer uptake in a bone scan; this is consistent with findings in other reported cases of melorheostosis [5, 10].

Melorheostosis is a rare disease; it is heterogeneous in its presentation, leaving some uncertainty in establishing the diagnosis. However, among monostotic sclerosing bone diseases, the irregular cortical hyperostosis is specific for this condition.

In conclusion, this is the first report indicating that denosumab has a place in the treatment of melorheostosis when the effect of bisphosphonate treatment is insufficient.

## Conclusion

### The rationale for the conclusion

In this case of monostotic melorheostosis, there was a recurrence of disabling pain after one injection with zoledronic acid and lack of effect with repeated administrations.

To halter the increased bone turnover a more powerful anti-reabsorptive agent was chosen with the RANKL inhibitor denosumab. In this case, there was pain relief for 6 weeks after the first administration. At this time the pain intensity increased again, with a slight increase in bone markers. One could speculate if this was indicative of an underlying state of increased consumption of osteoprotegerin. It was decided to shorten the interval between administrations to 8 weeks resulting in continuous pain remission for the following 8 months. The stationary findings on bone scintigraphy after 1.5 years of treatment might be explained by the size of the affected area, and thus we expect decreased activity in future scans.

### The primary “take-away” lesson from this case report

This is the first report indicating that denosumab has a place in the treatment of melorheostosis when the effect of bisphosphonate treatment is insufficient.
